# A pilot study on improvements in attention function in major depressive disorder after 12 weeks of escitalopram monotherapy or combined treatment with agomelatine

**DOI:** 10.3389/fpsyt.2023.1188175

**Published:** 2023-06-22

**Authors:** Zhe Li, Ting-Ting Wu, Yi-Ting Xiong, Xin-Yang Zhang, Yan-Ping Bao, Li-Bo Guo, Bao-Jie Han, Su-Xia Li, Yu-Feng Wang, Lin Lu, Xue-Qin Wang

**Affiliations:** ^1^Peking University Sixth Hospital, Peking University Institute of Mental Health, NHC Key Laboratory of Mental Health (Peking University), National Clinical Research Center for Mental Disorders (Peking University Sixth Hospital), Peking University, Beijing, China; ^2^Beijing Key Lab of Learning and Cognition, School of Psychology, Capital Normal University, Beijing, China; ^3^National Institute on Drug Dependence and Beijing Key Laboratory of Drug Dependence Research, Peking University, Beijing, China; ^4^Peking-Tsinghua Centre for Life Sciences and Peking University-International Development Group, McGovern Institute for Brain Research, Peking University, Beijing, China

**Keywords:** major depressive disorder, attentional networks test, escitalopram, agomelatine, cognitive function, efficiency, executive control of attention, logical memory

## Abstract

**Objective:**

This study aimed to explore both impairments in attention function in patients with major depressive disorder (MDD) and the efficacy of escitalopram monotherapy or combination therapy with agomelatine.

**Methods:**

A total of 54 patients with MDD and 46 healthy controls (HCs) were included. Patients were treated with escitalopram for 12 weeks; those who presented with severe sleep impairments were also given agomelatine. Participants were evaluated using the Attention Network Test (ANT), which included tests of alerting, orienting, and executive control networks. Concentration, instantaneous memory, and resistance to information interference were tested using the digit span test, and the logical memory test (LMT) was used to evaluate abstract logical thinking. The Hamilton Depression Rating Scale−17 items, Hamilton Anxiety Rating Scale, and Pittsburgh Sleep Quality Index were used to assess depression, anxiety, and sleep quality, respectively. Patients with MDD were assessed at the end of weeks 0, 4, 8, and 12. HCs were assessed once at baseline.

**Results:**

Compared with HCs, patients with MDD showed significantly different alerting, orienting, and executive control functions of attention networks. Treatment with escitalopram alone or combined with agomelatine significantly improved LMT scores at the end of weeks 4, 8, and 12 and restored scores to the level of HCs at the end of week 8. Total Toronto Hospital Test of Alertness scores in patients with MDD increased significantly after 4 weeks of treatment. The ANT executive control reaction time in patients with MDD decreased significantly after 4 weeks of treatment, with this decrease lasting until the end of week 12, but scores did not return to the levels of HCs. Combined treatment with escitalopram and agomelatine led to more improvement in ANT orienting reaction time and was accompanied by a greater reduction of total scores on the Hamilton Depression Rating Scale−17 items and Hamilton Anxiety Rating Scale compared with escitalopram monotherapy.

**Conclusions:**

Patients with MDD showed overall impairments in three domains of attention networks as well as the LMT and a test of subjective alertness. Escitalopram monotherapy significantly improved the LMT scores and the executive control function scores in the ANT at the end of the fourth week of treatment, and the improvement was more extensive with combined escitalopram and agomelatine treatment.

## 1. Introduction

Major depressive disorder (MDD) is an affective psychiatric disorder characterized by significant and persistent depression, often accompanied by loss of interest, diminished ability to think or concentrate, and sleep disturbances. MDD has a high rate of suicide and recurrence, which brings a heavy emotional and financial burden to patients and their families (1). According to the World Health Organization, MDD is expected to be the second most disabling and life-burdening disease in the world by 2030 (2). In addition, the prevalence of mental illness in Chinese adults is now as high as 17.5%, with MDD being the most common mental disorder (3).

Patients with MDD commonly experience impairments in cognitive functions, including executive function, learning and memory, processing speed, and attention (4, 5), which greatly affects their daily life and work. Notably, attentional deficits in MDD have been shown to be associated with higher relapse rates and poorer outcomes (4, 6, 7). Patients with MDD exhibit greater distractibility, an inability to sustain attention, and an inability to multitask (6, 8, 9). Previous studies revealed that attention has three separable networks that contribute to the maintenance of readiness (alerting), selecting and moving attention to stimuli (orienting), and resolving conflicts and coordinating among thoughts and actions (executive control) (10–12). Alerting is defined as achieving and maintaining an alert state, orienting is the selection of information from sensory inputs, and executive control is the resolution of conflict among responses. The three attention networks are independent of each other and have specific neuroanatomical and neurobiochemical mechanisms, but they are, to some extent, interconnected and work together to complete information processing.

Previous studies have suggested that alerting is associated with the cortical distribution of norepinephrine (13), orienting is associated with frontal and parietal acetylcholine (14, 15), and executive control is associated with the mesocortical dopamine (DA) pathway (16). However, serotonin and norepinephrine reuptake inhibitors and alpha-2 adrenergic receptor agonists have been shown to improve selective (17) and sustained attention in some studies (18, 19), but the results have been mixed (20, 21). Escitalopram is an SSRI with a high selectivity for serotonin re-uptake (22) and has been shown to significantly improve verbal, nonverbal, and working memory in patients with MDD (23, 24). Serotonin has regulatory effects on the DA system (25) and, according to the “affect-as-information” framework (26), emotional/mood states can influence attention and overall cognitive styles. Thus, escitalopram may contribute to improvements in attention function. In addition, more than 90% of patients with MDD simultaneously have varying degrees of sleep problems (27), and cognitive functions in these patients are severely negatively affected by poor sleep quality (28). Sedative and hypnotic drugs can interfere with cognitive functions such as attention and memory (29). Agomelatine, an agonist of melatonin receptors (MT1 and MT2) and an antagonist of the serotonin 2C receptor (5-HT_2C_), can exert different effects at different stages of the diurnal cycle (30). During the daytime, the 5-HT_2C_ antagonism of the drug predominates, and vigilance arises (31). During the night, agomelatine produces sleep-promoting effects that exceed the vigilance effects (30). Through this double action, agomelatine promotes and maintains sleep and helps to maintain diurnal alertness (31).

We hypothesized that escitalopram, a typically prescribed SSRI for MDD, can significantly improve attention network function in patients with moderate or severe MDD. In the present study, the effects of a 12-week full-dose escitalopram treatment were compared with healthy controls (HCs). Further, patients with severe sleep quality impairments were treated with a combination of escitalopram and agomelatine to investigate the effect on sleep symptoms in patients with moderate or severe MDD.

## 2. Materials and methods

### 2.1. Participants

Patients with MDD were enrolled through outpatient clinics at Peking University Sixth Hospital after diagnosis based on the Diagnostic and Statistical Manual of Mental Disorders, 5th ed. (DSM-5) (32). Included patients had moderate or severe MDD without suicidal ideation. A group of healthy controls, matched with the MDD group for age, sex, and years of education, was recruited from the community via advertisement.

#### 2.1.1. Major depressive disorder group

The inclusion criteria were as follows: (1) met the DSM-5 diagnostic criteria of MDD, (2) age 18–50 years, (3) right-handed, (4) Hamilton Depression Rating Scale−17 items (HAMD-17) score ≥ 22, (4) Hamilton Anxiety Rating Scale (HAMA) score ≥ 14, (5) at least 5 years of education, and (6) the ability to understand and read Mandarin Chinese. The exclusion criteria included: (1) comorbidity with other mental disorders assessed by the Mini-International Neuropsychiatric Interview (33), English Version 7.0.2 (34), (2) suicidal ideation (HAMD-17 item 3 score > 2), (3) patients who had received any medical treatment, including traditional Chinese medicine treatment, within 1 month of study enrolment and treatments such as modified electroconvulsive therapy, repeated transcranial magnetic stimulation, or transcranial electrical stimulation within 6 months of study enrollment, (4) current diagnosis with significant sleep disorders except for insomnia, (5) alcohol use within 1 week prior to enrollment or tobacco use of more than five cigarettes per day, and (6) shift work or travel causing jet lag or the presence of social jet lag within 3 months of study enrollment.

#### 2.1.2. Healthy control group

HCs were matched with patients in the MDD group according to age, sex, and years of education. HCs had received a general health examination within 6 months of enrolment and had no medical conditions. All of the exclusion criteria for patients with MDD applied to the HCs, and HCs did not have a current or previous mental disorder as assessed by the Mini-International Neuropsychiatric Interview 7th ed., including MDD.

### 2.2. Tests and assessments

#### 2.2.1. Attention Network Test

The Attention Network Test (ANT) is an assessment tool developed by Fan et al. to explore and assess the efficiency of three separable attention networks (i.e., alerting, orienting, and executive control networks) (35). The alerting network is responsible for achieving and maintaining a state of alertness by altering one's internal state to prepare for perceiving and responding to stimuli from the external environment. The orienting network is responsible for the selection of information from sensory inputs. The executive control network is responsible for resolving conflicts between responses. The efficiency of each network was measured using differences in response time (RT) and error rates (ER) between different conditions (36).

**Figure 1:**
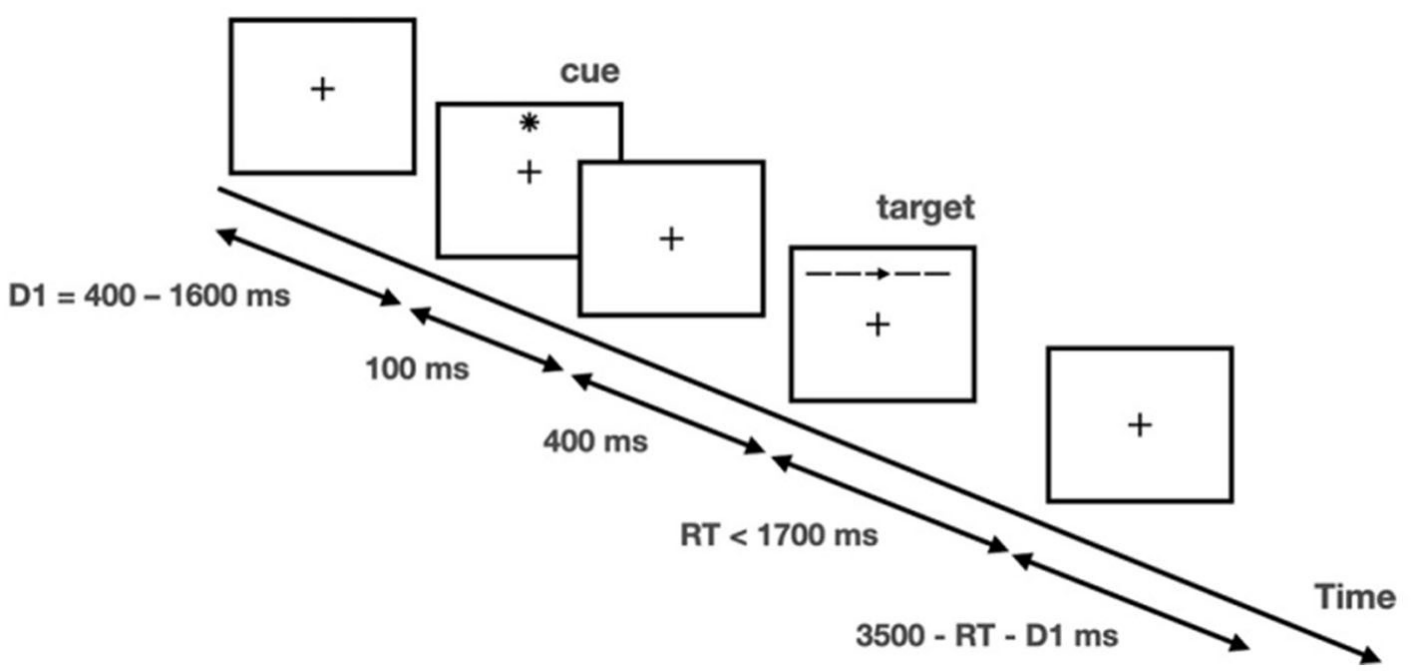
Illustration of attention network test. Adapted with permission from Fan et al. (35). Copyright 2002 Massachusetts Institute of Technology.

The ANT was programmed and run using E-prime software (v2.10) (37). A schematic of each trial is included in Figure 1. A fixation cross was presented at the center of a screen during the entire trial, and participants were required to maintain focus on the cross. There was a 400–1,600-ms fixation period at the beginning of each trial, and then an asterisk appeared for 100 ms as the cue. After a 400-ms fixation interval, a row of five stimuli was presented above or below the fixation cross, with a left/right-pointing arrow in the middle as the target and the other four stimuli, which would be four left/right-pointing arrows or four short horizontal bars, as the distractors. Participants were required to indicate the direction of the target by pressing the left or right arrow keys on a keyboard as quickly and accurately as possible. The response window was 1,700 ms starting at the onset of the arrows. There was a varied fixation period at the end of the trial, and the total trial duration per trial was fixed at 4,000 ms.

The task was given in a 4 (cueing type) × 3 (flanker type) factorial design. Cueing type refers to (1) no cue (no star sign displayed prior to arrow set); (2) center cue (star sign displayed at the center of the screen); (3) spatial cue (star sign displayed above or below the fixation cross to indicate the spatial location of the upcoming target; 100% valid); and (4) double cue (two star signs displayed respectively above and below the fixation cross to alert that the target would appear soon). Flanker type refers to (1) congruent (flanker arrows pointed in the same direction as the target); (2) incongruent (flanker arrows pointed in the opposite direction of the target); and (3) neutral (flankers were four bars). There were 26 trials in each of the 12 conditions, and the entire task consisted of 312 trials, with a total duration of approximately 30 min. Another 12 practice trials were provided before the formal test to familiarize the participants with the task.

### 2.3. Calculating the efficiency of attention networks

The RTs and ERs in each condition were computed. For each participant, the ERs for each condition were calculated as the proportion of trials with incorrect responses or no responses to the number of trials in that condition. The RT for each trial was calculated as the interval between the onset of the target and the button press. Trials with no responses or incorrect responses were excluded from the RT analysis. We further excluded trials with RTs exceeding three standard deviations of the mean RT (also known as overall RT; the average RT of all trials with correct reactions, regardless of network) in each condition, and the condition-wise mean RT across the remaining trials was then computed. Attention function was then computed as the difference between corresponding conditions in terms of RT and ER, as in Fan et al. (38). Specifically, the alerting effect was computed as the values of the no cue condition minus those of the double cue condition, with larger values indicating a higher efficiency of the alerting network. The orienting effect was computed as the values of the double cue condition minus those of the spatial cue condition, with larger values indicating higher efficiency of the orienting network. The conflict effect was computed as the values of the incongruent condition minus those of the congruent condition, with larger values indicating lower efficiency of the executive control network. In addition, we also computed the mean RTs and ERs across all conditions.

### 2.4. Memory assessments

The digit span test (DST) of the Wechsler Memory Scale (39) mainly measures concentration, instantaneous memory, and resistance to information interference. The logical memory test (LMT) of the Wechsler Memory Scale is a standardized assessment of narrative episodic memory that assesses the process of forming an understanding or conclusion through abstract logical thinking such as conceptual thinking, reasoning, analysis, and judgment after receiving external information, and the maintenance of these in episodic memory.

### 2.5. Clinical scales

The Toronto Hospital Test of Alertness (THAT) and the ZOGIM-A (Alertness Questionnaire) (40–42) were used to assess alertness function and influencing factors using self-reports. A lower total THAT score indicates impaired alertness. The ZOGIM-A assesses alertness throughout the day, measuring the effects of various environmental factors (e.g., caffeine, exercise) on subjective alertness and the proportion of the time spent at a high level of alertness. The THAT and the ZOGIM-A are not only applied to measure different aspects of alertness but can also be combined functionally (43). Depression, anxiety, and sleep quality were assessed using the HAMD-17 (44), the 14-item HAMA (45), and the Pittsburgh Sleep Quality Index (PSQI) (46), respectively. Intelligence quotients were measured using the Wechsler Adult Intelligence Scale (47). A general questionnaire was designed to collect demographic data, such as sex, age, years of education, and other information.

### 2.6. Study design and procedure

**Figure 2:**
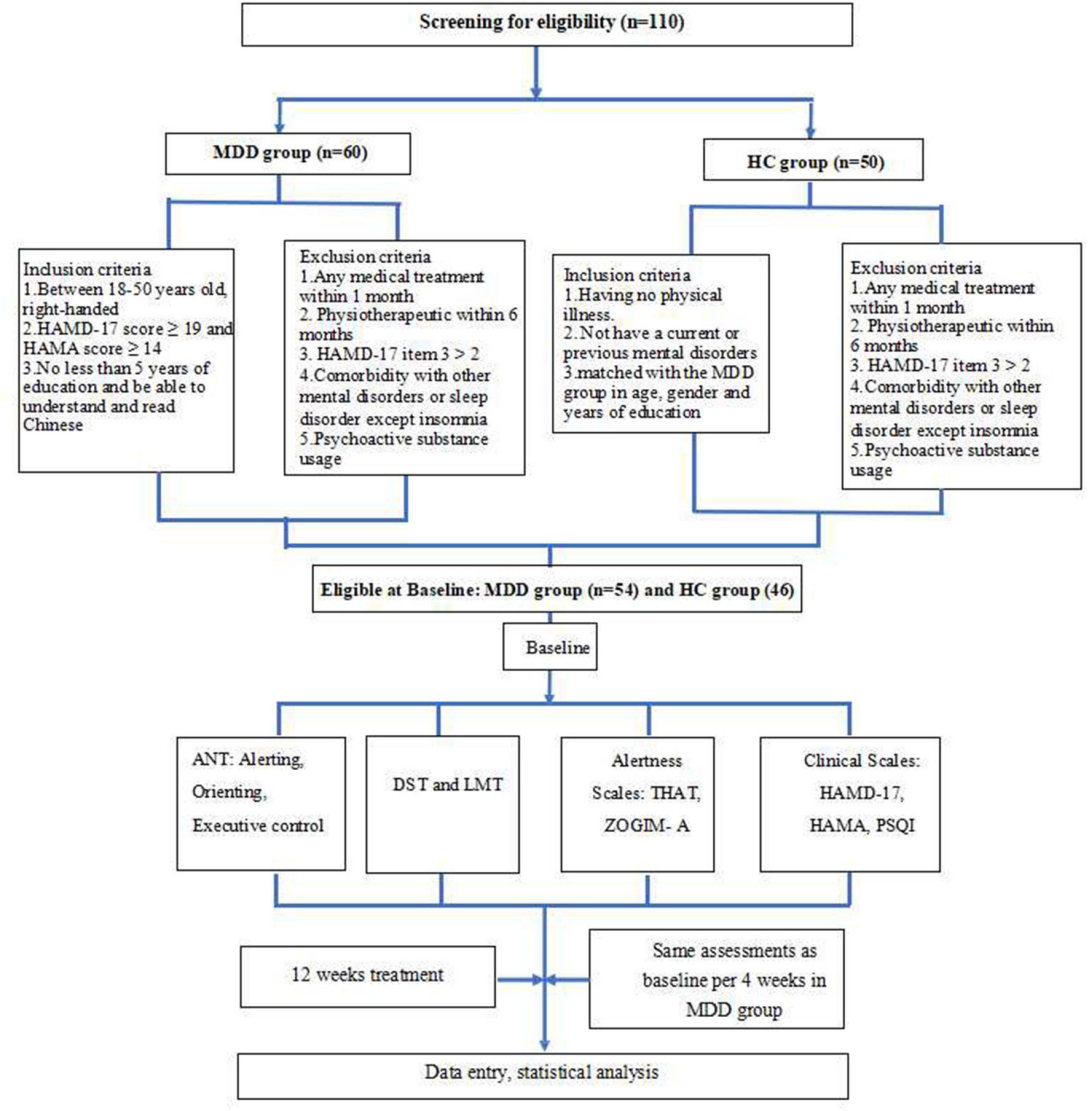
Flowchart of the study. MDD, major depressive disorder; HC, healthy control; HAMD, Hamilton Depression Rating Scale; HAMA, Hamilton Anxiety Rating Scale; ANT, Attention Network Test; DST, digit span test; LMT, Logical Memory test; THAT, Toronto Hospital Alertness Test; ZOGIM-A, Alertness Questionnaire; PSQI, Pittsburgh Sleep Quality Index.

Clinical assessments and an ANT evaluation of all participants were performed at baseline. Then, patients with MDD were given escitalopram alone for 12 weeks of treatment (open-label). The dose of escitalopram was 10–20 mg QD determined by a medical doctor. Patients with severely poor sleep were administered agomelatine (25 mg) to be taken before sleeping. For patients with MDD, all tests and evaluations were performed repeatedly at the end of the 4th, 8th, and 12th weeks of treatment. The response rate was measured as the total number of patients who had a reduction of more than 50% of their total HAMD-17 score (22), and clinical remission was defined as a HAMD-17 score of ≤ 7 (20). To further understand whether escitalopram combined with agomelatine was better for cognitive improvement, in addition to comparing patients with MDD with HCs, we also divided the MDD group into two subgroups of escitalopram treatment alone and combined treatment with agomelatine. Raters were single-blinded and conducted the ANT and clinical assessments separately from the treatment team. More details are shown in Figure 2.

This study was reviewed and approved by the Medical Ethics Committee of Peking University Sixth Hospital (Mental Health Institute) (Approval No. 2016-12), and all participants signed informed consent forms. The study protocol was registered at clinicaltrials.gov with the identification number NCT04978220.

### 2.7. Statistical analysis

Statistical analysis was performed using SPSS software (v20.0; IBM Corp., Armonk, NY, USA). Demographic characteristics were analyzed using descriptive statistics (mean and standard deviation or frequency). A scatterplot fitting analysis was used to find whether data of ANT parameters conformed to normal distribution. ANT parameters are expressed as median and quartiles (Q1, Q3). One-way analysis of variance was used to compare differences in clinical assessments between different time points (0, 4, 8, and 12 weeks). Considering possible variance, determined by homogeneity tests, ANT parameters were analyzed using the Mann-Whitney U test within the MDD group at different time points. The Mann-Whitney U test was also used to compare differences in ANT parameters between groups. Correlation coefficients were calculated using partial correlation analysis. *P* < 0.05 were considered statistically significant.

## 3. Results

### 3.1. Demographics and clinical assessments at baseline

#### 3.1.1. Demographics

In total, 54 of 60 patients with MDD and 46 of 50 HCs successfully enrolled at baseline. There were 38 (70.37%), 37 (68.52%), and 33 (61.11%) patients with MDD who completed 4, 8, and 12 weeks of treatment and all assessments, respectively. There were no significant differences in age, sex, years of education, or IQ between the HCs and the patients with MDD. In the MDD group, eight patients (14.81%) had recurrent major depressive episodes. The duration of the current depression episode was 5.98 ± 4.79 months in the 54 patients with MDD measured at baseline (Table 1).

**Table 1:** The demographic characteristics of patients with major depressive disorder and healthy controls at baseline.

**Variable**	**MDD (n = 54)**		**HCs (n = 46)**		***t*/*χ^2^***	***P***
	**Mean**	**SD**	**Mean**	**SD**		
Age (years)	31.06	9.41	30.04	7.20	−0.60	0.553*^*a*^*
**Gender**						
Male (n [%])	18 [33.33]		16 [34.78]		0.02	0.879*^*b*^*
Female (n [%])	36 [66.67]		30 [65.22]			
Education (years)	14.91	3.38	14.83	4.06	−0.11	0.913*^*a*^*
IQ	120.98	14.61	118.87	22.47	−0.55	0.586*^*a*^*
Recurrent MDE (n [%])	8 [14.81]					
Duration of the current MDE (months)	5.98	4.79				

^a^Independent Samples t-test, ^b^chi-square test. The values are expressed as numbers (%), mean ± standard deviation. MDD, major depressive disorder; HC, healthy control; SD, standard deviation; IQ, intelligence quotient; MDE, major depressive episode.

#### 3.1.2. Assessment scales and memory tests

The total THAT scores were significantly lower in the MDD group compared with the HC group (*t* = 10.47, *P* < 0.01), suggesting that there was an impaired level of alertness in patients with MDD. However, differences in the total ZOGIM-A scores between the groups were not significant (*t* = 0.52, *P* > 0.05), indicating that subjective alertness levels of the patients with MDD and the HCs were not affected by environmental factors (e.g., caffeine, exercise). Meanwhile, the logical memory of the patients with MDD was impaired, and their LMT error scores were significantly higher than those of the HCs (*t* = −3.12, *P* < 0.01). The patients with MDD included in the present study had moderate to severe depressive episodes (HAMD-17 = 25.76 ± 5.43) accompanied by symptoms of anxiety (HAMA = 22.54 ± 6.14) and poor sleep quality (PSQI = 13.41 ± 4.14) (Table 2). Only one patient with MDD had no sleep disturbances according to the PSQI (total score < 5).

**Table 2:** Clinical assessments of patients with major depressive disorder and healthy controls at baseline.

**Variable**	**MDD (n = 54)**		**HCs (n = 46)**		***t***	***p^*a*^***
	**Mean**	**SD**	**Mean**	**SD**		
THAT	13.37	6.90	29.54	8.32	10.47	0.000
ZOGIMA	34.76	5.27	35.39	6.92	0.52	0.606
DST	14.26	3.67	14.70	3.17	0.63	0.529
LMT	5.17	4.03	3.07	2.66	−3.12	0.002
HAMD	25.76	5.43	0.96	0.99	−32.96	0.000
HAMA	22.54	6.14	1.15	0.89	−25.27	0.000
PSQI	13.41	4.14	3.61	2.59	−14.40	0.000

^a^Independent Samples t-test. The values are expressed as mean ± standard deviation. MDD, major depressive disorder; HC, healthy control; SD, standard deviation; THAT, Toronto Hospital Alertness Test; ZOGIM-A, Alertness Questionnaire; DST, digit span test; LMT, Logical Memory test; HAMD, Hamilton Depression Rating Scale; HAMA, Hamilton Anxiety Rating Scale; PSQI, Pittsburgh Sleep Quality Index.

#### 3.1.3. Attention Network Test

The patients with MDD showed significant impairments in all three attentional networks compared with HCs, as detailed in Table 3. The patients with MDD showed significantly longer RTs in the alerting network (*Z* = −4.65, *P* < 0.01) and the executive control network (*Z* = −6.09, *P* < 0.01) but showed shorter RTs in the orienting network (*Z* = −5.99, *P* < 0.01) compared with those in the HC group. Compared with the HCs, the patients with MDD also showed significantly higher ERs in the orienting network (*Z* = −2.00, *P* < 0.05) and significantly higher mean ERs (*Z* = −6.23, *P* < 0.01). The miss rate was higher in the patients with MDD compared with that in the HCs (*Z* = −6.55, *P* < 0.01) at baseline. These findings suggested that patients with moderate to severe MDD accompanied by anxiety and poor sleep quality had significant overall deficits in attention networks.

**Table 3:** Attention Network Test parameters in patients with major depressive disorder and healthy controls at baseline.

**Variable**	**MDD (n = 54)**		**HCs (n = 46)**		***Z***	***p^*a*^***
	**Median**	**Quartile (Q1, Q3)**	**Median**	**Quartile (Q1, Q3)**		
**RT (msec)**						
Alert	40.02	(19.96, 61.13)	0.00	(0.00, 0.04)	−4.65	0.000
Orienting	22.82	(3.62, 53.24)	84.24	(49.25, 118.58)	−6.09	0.000
Conflict	115.24	(79.53, 141.96)	40.56	(22.65, 88.80)	−5.99	0.000
Mean RT	612.37	(534.80, 662.94)	619.43	(560.81, 685.89)	−0.39	0.699
**ER (%)**						
Alert	0.00	(−2.08, 2.08)	0.00	(−0.52, 2.60)	−1.58	0.115
Orienting	0.00	(0.00, 2.08)	0.00	(−1.69, 2.08)	−2.00	0.046
Conflict	2.78	(0.00, 4.51)	2.08	(0.00, 5.73)	−0.50	0.614
Mean RT	1.39	(0.69, 3.21)	35.59	(5.43, 63.15)	−6.23	0.000
Miss rate	0.00	(0.00, 0.01)	0.69	(0.03, 2.17)	−6.55	0.000

^a^Mann-Whitney test. The values are expressed as medians, quartiles (Q1, Q3); MDD, major depressive disorder; HC, healthy control; ANT, Attention Network Test; RT, reaction time; ER, error rate.

### 3.2. Clinical assessments during treatment in patients with major depressive disorder

**Figure 3:**
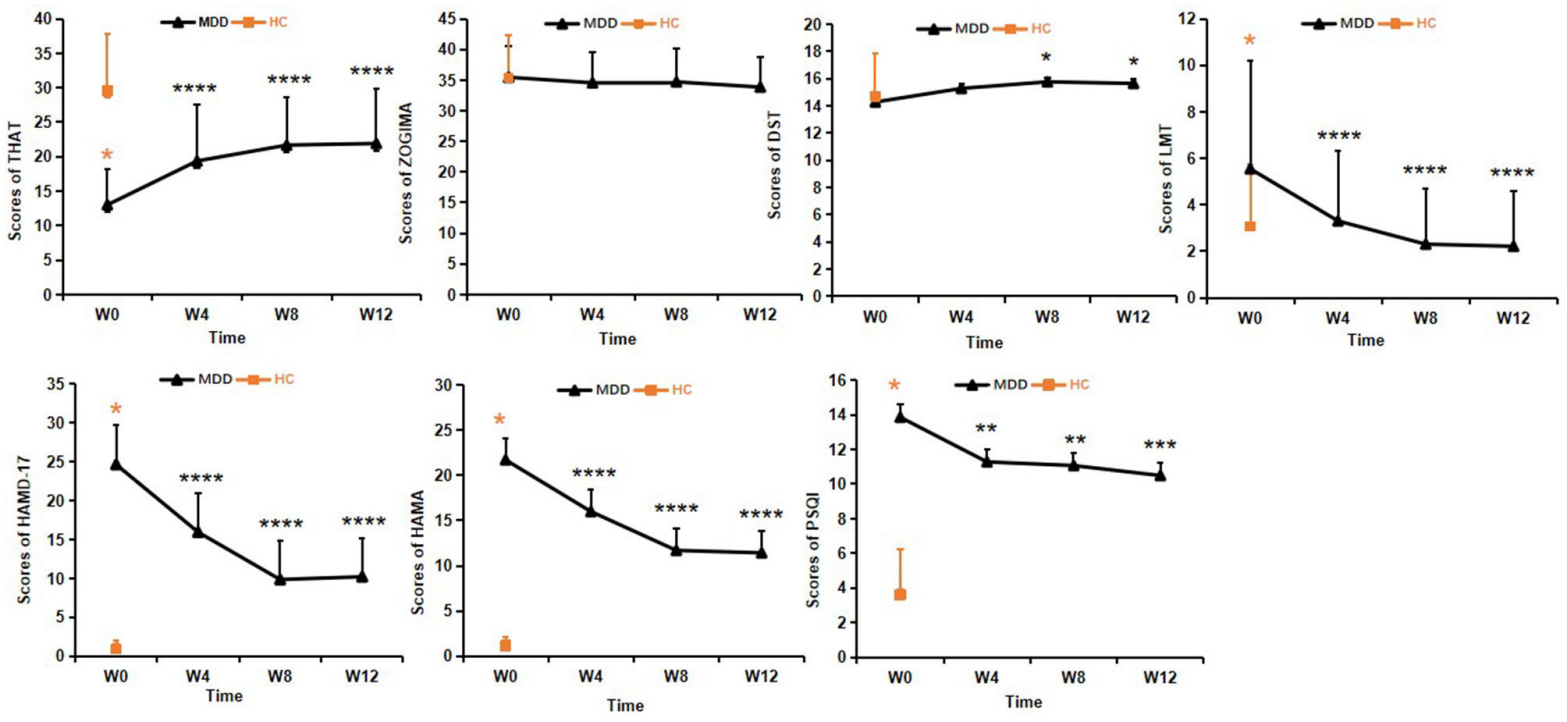
Clinical assessments in the MDD group during the 12-week treatment course. One-way analysis of variance was used to test differences. ^*^p < 0.05, ^**^p < 0.01, ^***^p < 0.001, ^****^p < 0.0001; values are expressed as mean ± standard deviation. THAT, Toronto Hospital Alertness Test; ZOGIM-A, Alertness Questionnaire; DST, digit span test; LMT, Logical Memory test; HAMD, Hamilton Depression Rating Scale; HAMA, Hamilton Anxiety Rating Scale; PSQI, Pittsburgh Sleep Quality Index.

As shown in Figure 3, there were significant differences in total THAT scores between baseline and measurements at 4, 8, and 12 weeks of treatment (*F*_3, 96_ = 18.35, *P* < 0.01). *Post hoc* least significant differences (LSD) comparisons of the THAT scores revealed a significant improvement at weeks 4, 8, and 12 compared to baseline (*P* < 0.001), and no significant differences were found among the scores at weeks 4, 8 and 12 of treatment (*P* > 0.05). The results indicated that subjective alertness in the patients with MDD improved after 4 weeks of treatment and these levels were sustained until the end of the 12-week treatment course (Figure 3).

No significant changes in the ZOGIM-A total scores were found after treatment (*F*_3, 96_ = 1.13, *P* > 0.05), suggesting that subjective alertness levels were not influenced by environmental factors (e.g., caffeine, exercise) in the patients with MDD. Improvements in alertness assessed by THAT might have been due to antidepressant treatment (Figure 3).

As for the DST scores, significant changes in the total scores were found between time points (*F*_3, 96_ = 2.89, *P* < 0.05). After 4 weeks of treatment, the DST scores showed significant improvement from baseline (*P* < 0.05) in the MDD group, but the DST scores were not significantly different at baseline (*P* > 0.05) between the MDD and HC groups (Figure 3).

Changes in the LMT scores from baseline to weeks 4, 8, and 12 showed statistical significance (*F*_3, 96_ = 20.57, *P* < 0.01). Post hoc LSD comparison revealed continuous improvement from week 4 to week 12 (all *P* < 0.0001), and there were no significant differences between the LMT scores at weeks 8 and 12 within the MDD group (*P* > 0.05), There were no significant differences between the MDD and HC groups at the end of 4 weeks treatment (*P* > 0.05). This suggested that improvement in logical memory appeared after 4 weeks of treatment and the LMT scores were restored to the levels of HCs at 8 weeks and were sustained until the end of the 12-week treatment course (Figure 3).

After 8 weeks of treatment, the remission rate was 33.33% and the response rate was 48.15% in the patients with MDD. Among the 33 patients with MDD who completed the 12-week treatment course, the remission rate was 48.65% and the response rate was 70.27%.

One-way analysis of variance was performed to analyze all clinical assessments. The total scores of both HAMD-17 (*F*_3, 96_ = 59.50, *P* < 0.01) and HAMA (*F*_3, 96_ = 33.241, *P* < 0.01) were significantly lower at the end of the 12-week treatment in the MDD group compared with those at baseline, and *post hoc* LSD tests showed that the total scores of both HAMD-17 and HAMA continuously decreased until the end of the measurement period (*P* < 0.0001) (Figure 3).

The changes in the total PSQI scores in the MDD group between baseline, 4, 8, and 12 weeks were statistically significant (*F*_3, 96_ = 9.054, *P* < 0.01). *Post hoc* LSD comparison revealed significant improvement in sleep quality at 4, 8, and 12 weeks compared to baseline (*P* < 0.01), and no significant differences were found between scores at weeks 8 and 12. There were no significant differences of PSQI scores among 4, 8 and 12 weeks of treatment (*P* > 0.05). These results indicated that sleep quality in patients with MDD improved after 4 weeks of treatment and this improvement was sustained until the end of the measurement period 12 weeks (Figure 3).

### 3.3. Attention Network Test scores during treatment in patients with major depressive disorder

A scatterplot fitting analysis showed that ANT parameters were linearly distributed at every measurement point. However, different parameters across the four measurements were not linearly distributed. Therefore, nonparametric analysis was adopted to analyze changes in ANT parameters.

**Figure 4:**
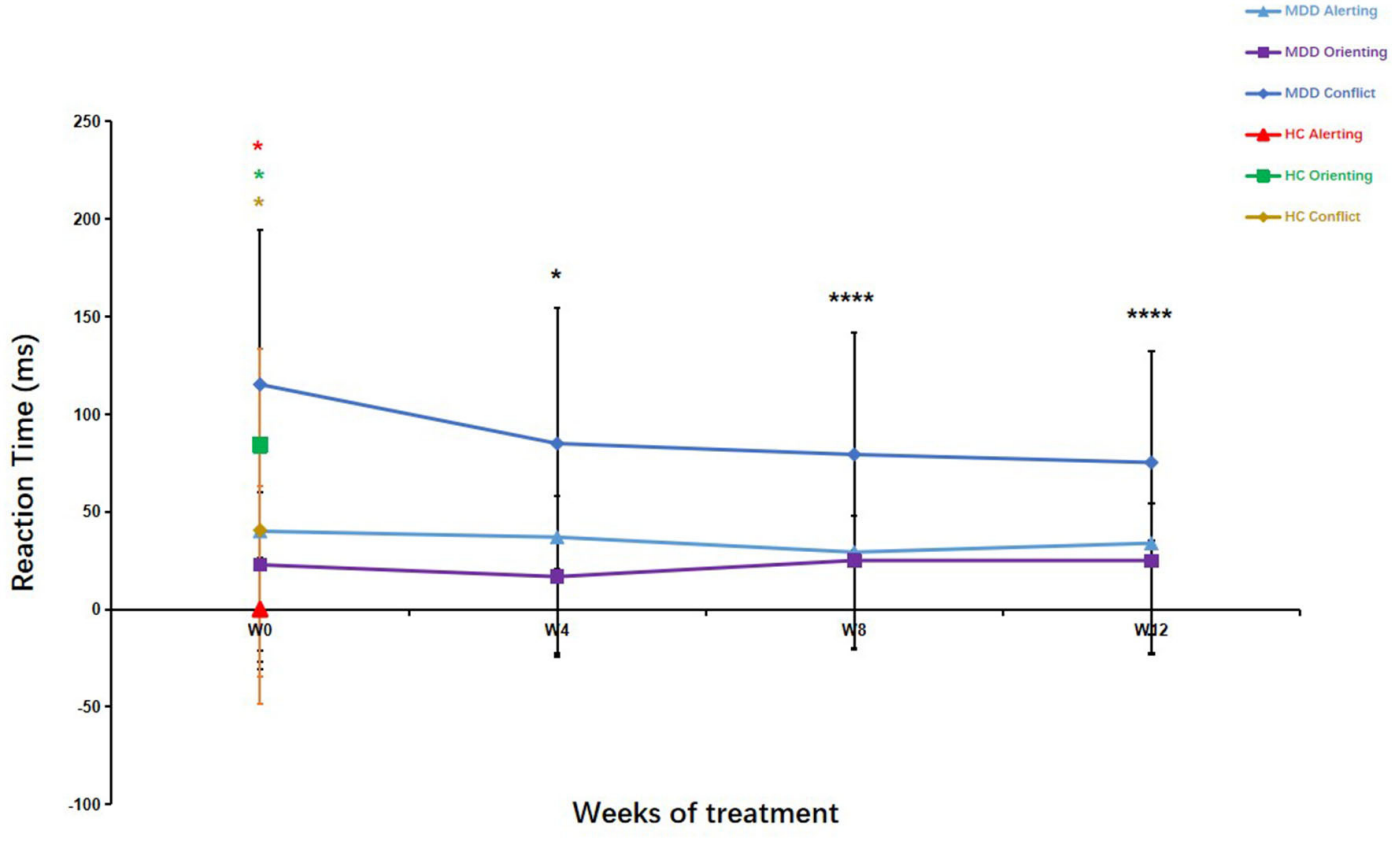
Attention Network Test (ANT) parameters in patients with major depressive disorder over the 12-week treatment course. Mann–Whitney U test was used to test differences between parameters. ^*^p < 0.05, ^****^p < 0.0001; values are expressed as median, quartiles (Q1, Q3). Error bars represent Q1–Q3.

As shown in Figure 4, the patients with MDD had significantly longer alerting (*Z* = −4.65, *P* < 0.01) and conflict (*Z* = −6.09, *P* < 0.01) RTs and shorter orienting (*Z* = −5.99, *P* < 0.01) RTs compared with the HCs. The conflict RTs showed significant differences over the 12 weeks of treatment (χ^2^= 21.10, df = 3, *P* < 0.01). The conflict RTs improved significantly after 4, 8, and 12 weeks of treatment compared with those at baseline (4 weeks, *Z* = −2.18, *P* < 0.05; 8 weeks, *Z* = −3.56, *P* < 0.01; 12 weeks, *Z* = −3.92, *P* < 0.01). There were no significant differences between conflict RTs at weeks 4, 8, and 12 (4 weeks vs. 8 weeks, *Z* =-1.06, *P* > 0.05; 4 weeks vs. 12 weeks, *Z* = −1.70, *P* > 0.05; 8 weeks vs. 12 weeks, *Z* = −0.81, *P* > 0.05). Conflict RTs after 12 weeks of treatment in the MDD group were significantly lower compared with those at baseline in the HCs (*Z* = −4.658, *P* < 0.01). Regarding alerting and orienting RTs, there were no significant differences between different measurement points in the MDD group (alertness, *Z* =-0.27, *P* > 0.05; orienting, *Z* = −0.88, *P* > 0.05) (Figure 4).

The mean RTs were statistically different (8 weeks, *Z* = −2.00, *P* < 0.05; 12 weeks, *Z* = −2.32, *P* < 0.05) in the MDD group after 8 and 12 weeks of treatment compared to baseline, but there was no significant difference after 4 weeks of treatment (*Z* = −1.77, *P* > 0.05). Similarly, there was a significant difference between the patients with MDD at the end of the 12-week treatment course and the HCs (at baseline) in mean RT (*Z* = −2.395, *P* < 0.05). These results suggested that the executive control network in the patients with MDD did not recover to the levels seen in the HCs after 12 weeks of treatment.

The ERs of the orienting network were higher in the MDD group than in the HC group (*Z* = −2.00, *P* < 0.05). However, no differences in the ERs of both the alerting network (*Z* = −1.58, *P* > 0.05) and the executive control network (*Z* = −0.50, *P* > 0.05) were found between the two groups. The mean ERs of the three networks in the MDD group were higher than those in the HC group (*Z* = −6.23, *P* < 0.01).

To clarify whether improvements in sleep quality could further help improve cognitive function, we divided patients with MDD into two subgroups (escitalopram treatment alone [29 patients] and combination with agomelatine [25 patients]). At baseline, there were no significant differences between the subgroups in demographic data and clinical assessment scores (Supplementary Tables S1–S3). There were significant differences in the HAMD-17 (*t* = 2.91, *P* < 0.01) and HAMA (*t* = 2.40, *P* < 0.05) scores between the two subgroups after 4 weeks of treatment. However, there was no significant difference in the total PSQI scores between the two subgroups (*t* = −1.05, *P* > 0.05). There were also no statistically significant differences in the THAT, ZOGIM-A, DST, or LMT scores between the two subgroups after 4 weeks of treatment (*P* > 0.05; Supplementary Table S4).

As shown in Supplementary Table S5, compared with the escitalopram alone subgroup, escitalopram combined with agomelatine had better effects on the improvement of the orienting RTs (*Z* = −1.98, *P* < 0.05) after 4 weeks of treatment. The differences in the conflict RTs (*Z* = −0.23, *P* > 0.05) and the alerting RTs (*Z* = −1.49, *P* > 0.05) between the two subgroups were not significant after 4 weeks of treatment. There were no significant changes in all the ERs (all *P* > 0.05) between the subgroups (Supplementary Table S5).

### 3.4. Correlation analysis after 4 weeks of treatment

**Figure 5:**
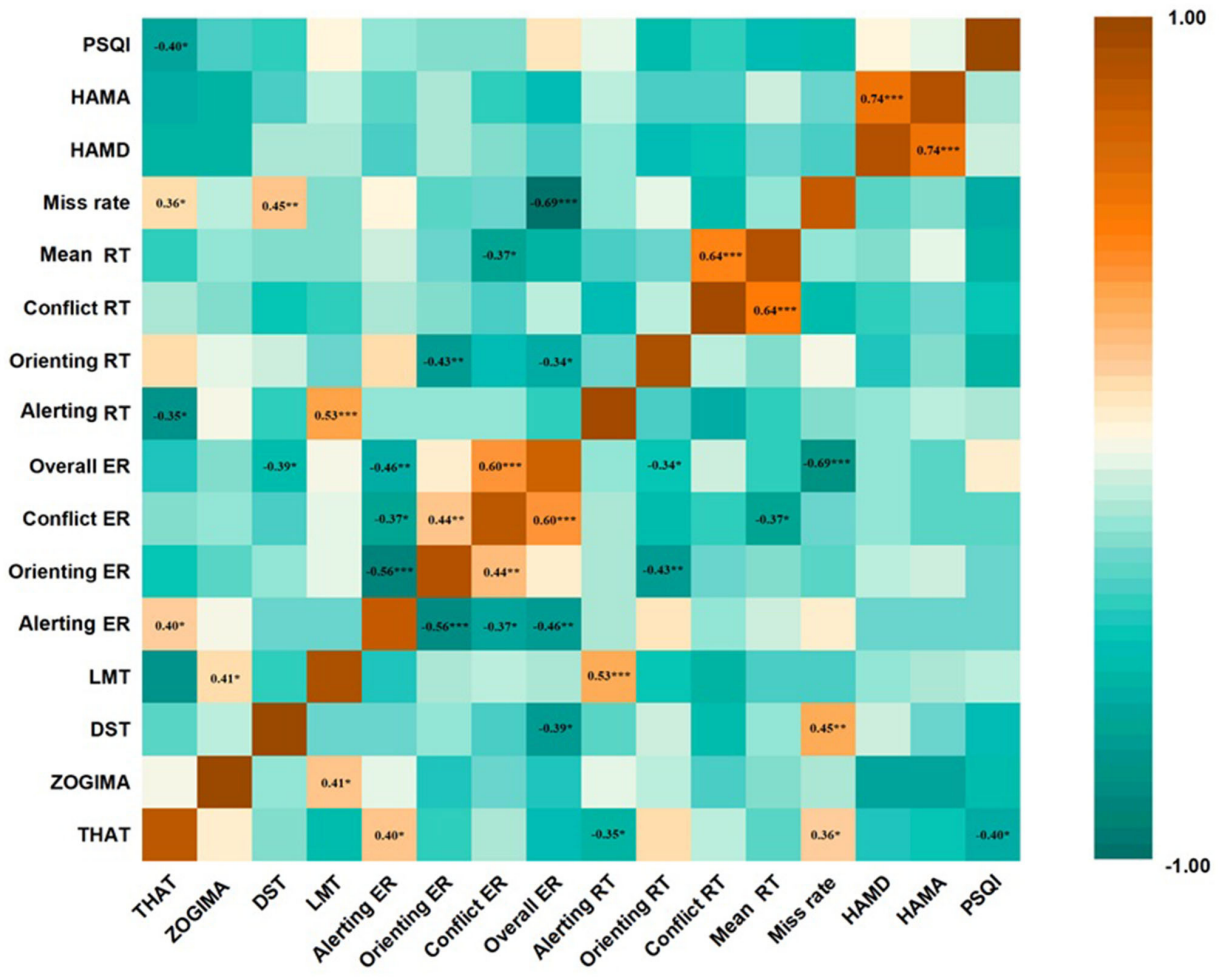
Correlation between changes in Attention Network Test (ANT) parameters and changes in clinical scale assessments in patients with major depressive disorder (MDD) at the end of the 4-week treatment course. Partial correlation analysis was performed for all correlations. ^*^p < 0.05, ^**^p < 0.01, ^***^p < 0.001. values are expressed as partial correlation coefficients. THAT, Toronto Hospital Alertness Test; ZOGIM-A, Alertness Questionnaire; DST, digit span test; LMT, logical memory test; HAMD, Hamilton Depression Rating Scale; HAMA, Hamilton Anxiety Rating Scale; PSQI, Pittsburgh Sleep Quality Index; RT, reaction time; ER, error rate.

Figure 5 shows the partial correlation analysis of changes in each ANT network and clinical parameter after 4 weeks of treatment. The reduction in the orienting RTs was negatively correlated with the reduction in the orienting ERs in the patients with MDD (r = −0.43, *P* < 0.05) as well as with the reduction in the mean ERs in the ANT (r = −0.34, *P* < 0.05), implying a potential speed-accuracy trade-off for the orienting network. There was no significant correlation between changes in the orienting RTs and changes in all clinical assessments, namely the HAMD-17, HAMA, PSQI, THAT, ZOGIM-A, DST, and LMT (*P* > 0.05); therefore, clinical improvements might not have contributed to reductions in the orienting RTs after 4 weeks of treatment. The reduction in the alerting RTs was positively correlated with the reduction in the LMT scores (r = 0.53, *P* < 0.05) and negatively correlated with the reduction in the THAT scores (r = −0.308, *P* < 0.05). The reduction in the conflict RTs was only positively correlated with the reduction of the ANT mean RTs (r = 0.64, *P* < 0.05), and all the other clinical assessment changes were not significantly correlated with the conflict RTs (*P* > 0.05).

The reduction in the alerting ERs positively correlated with the improvement of the HAMD-17 total scores (r = 0.24, *P* < 0.05), and the reduction in the orienting ERs negatively correlated with the reduction in the HAMD-17 total scores (r = −0.25, *P* < 0.05) after 4 weeks of treatment. The reduction in the alerting ERs and orienting ERs were positively (r = 0.243, *P* = 0.017) and negatively (r = −0.251, *P* = 0.013) correlated, respectively, with reductions in the psychomotor retardation item scores of the HAMD-17 after of 4 weeks of treatment. However, there was no significant correlation between the changes in the ANT parameters and the PSQI total scores; the miss rate of the ANT was moderately negatively correlated with the daytime function changes of the PSQI (item 7) (r = −0.53, *P* < 0.01).

## 4. Discussion

Patients with moderate and severe MDD showed significant abnormalities in the three attention networks, such as lower alertness and executive control function accompanied by more errors in sustaining attention. Treatment with escitalopram alone or combined with agomelatine significantly improved executive control function measured by the ANT in the patients with MDD after 4 weeks of treatment, and this effect lasted until the end of the present 12-week treatment course; however, these levels were not restored to normal levels. Compared with escitalopram monotherapy, combination treatment with agomelatine showed better improvement in the ANT orienting RTs accompanied by better improvement in the HAMD-17 and HAMA scores. Treatment with escitalopram alone or combined with agomelatine significantly improved the THAT and LMT scores of the patients with MDD after 4 weeks of treatment, and the LMT scores were restored to normal levels after 8 weeks of treatment. Changes in all the assessment scale scores had no significant correlation with the RTs in the ANT, but the reduction in the ANT alerting RTs was negatively correlated with the reduction of the THAT scores and positively correlated with the reduction of the LMT scores after 4 weeks of treatment.

Previous studies (4, 5) have reported impairments in cognitive function in patients with MDD, but few studies have been conducted specifically in patients with moderate and severe MDD. In the present study, no significant differences were observed between the patients with MDD and HCs in the ZOGIM-A scores at baseline, indicating that subjective alertness in patients with moderate (HAMD-17 scores between 19 and 22) and severe (HAMD-17 scores ≥ 23) MDD may be less affected by psychological factors, behavioral factors, and other factors (43). At the same time, the present findings indicated that the obvious and extensive impairment of attention networks in patients with MDD might be a specific biological characteristic. The alerting and conflict control RTs were significantly longer and the orienting RTs were significantly shorter in the patients with MDD compared with the HCs, suggesting that patients with moderate and severe MDD have lower alertness levels, less ability to sustain attention, and poorer executive function compared with HCs. Moreover, the ERs of the orienting network, but not those of the alerting and conflict control networks, were also higher in the MDD group than in the HC group. Interestingly, antidepressant treatment significantly improved the conflict RTs after 4 weeks of treatment and sustained these until the end of the 12-week measurement period, but this was not true for the alerting and orienting RTs in the ANT. Previous studies have shown that executive control of attention is supramodal, a mechanism that acts irrespectively of sensory input, and efficiently coordinates mental operations across modalities (48, 49). The executive control of attention, a high-level supervisory entity, coordinates thoughts and actions and efficiently allocates attentional resources to cross-modal relevant inputs and suppresses irrelevant information in further processing (49). This means that the executive control of attention may play a key role in the whole attention network. Longer conflict control RTs in the ANT indicates a lower efficiency of executive control of attention. This is in line with the present findings in patients with MDD, which suggest that the longer conflict control RTs might be a core characteristics of attention network function (19).

Although no significant differences were seen between the patients with MDD and HCs in the ERs of both the alerting and executive control networks, the ERs of the orientation network were significantly higher in the patients with MDD compared with those in the HCs; this contributed to a higher mean ER of the three networks in the patients with MDD and might be the result of shorter orienting RTs, showing that the higher likelihood of diversion of attention might increase task mistakes in patients with MDD. The lack of significant differences in the alerting and conflict ERs in the patients with MDD may have resulted from the compensatory effects of longer RTs in the ANT. In view of this, we performed a correlation analysis between the scores of psychomotor retardation items on the HAMD-17 and the changes in the ANT parameters. Reductions in the alerting ERs and increases in the orienting ERs were correlated with improvements in psychomotor retardation symptoms. These findings suggested that the improvement of subjective reaction speed resulted from alertness accuracy. However, the finding that orientation correction was negatively correlated with the improvement of psychomotor retardation symptoms needs to be comprehensively explored in future studies. Additionally, we also found that the miss rate was higher in patients with MDD than in HCs, indicating multi-dimensional cognitive impairment in patients with MDD.

Regarding logical memory, we found that treatment with escitalopram alone and combined with agomelatine significantly improved the LMT scores of the patients with MDD after 4 weeks, and scores were restored to those of the HCs after 8 weeks of treatment. Semkovska et al. found that patients with MDD had impairments in logical and working memory during both phases of active symptoms and remission, and logical memory impairments were more severe (50). Mendes et al. (51) also found that logical memory scores were lower in patients with MDD than in HCs, and logical memory scores did not recover to normal levels at the time of initial remission after antidepressant treatment (52). Interestingly, the present results showed that the LMT scores improved at 4 weeks of treatment and returned to the levels of the HCs after 8 weeks of treatment in the patients with MDD. These findings are not consistent with previous studies in terms of restoration to normal levels of logical memory ability. Given that the DST scores of the patients with MDD at baseline were not different from those of the HCs, the patients with MDD in the present study did not show impairments in instantaneous memory. In addition, we did not evaluate working memory in patients with MDD, and the clinical characteristics of the patients and the tools used to evaluate logical memory were different between previous studies and the present study.

Despite changes in the THAT scores suggesting a marked improvement in subjective alertness levels after treatment, with the improvements lasting until the end of the 12-week measurement period, these did not recover to the levels seen in the HCs. At the same time, reductions in the ANT alerting RTs were negatively correlated with reductions in the total THAT scores, suggesting that subjective and objective tests of alertness showed consistent results after 4 weeks of treatment. Regarding the ANT, the conflict RTs improved significantly in the MDD group after 4 weeks of treatment, and these improvements lasted until the end of the 12-week measurement period. However, these also did not recover to the levels seen in the HCs; the RTs of the orienting and alerting networks did not improve significantly at any measurement point in the treatment. Compared to other SSRIs, the effects of escitalopram are thought to purely result from the reuptake of serotonin (53, 54), which may theoretically have a negative effect on attention functions (11). Unexpectedly, escitalopram was shown to improve working memory, attention, and executive function as effectively as duloxetine (23) and led to a significant improvement in attention function in the treatment of attention-deficit/hyperactivity disorder in children and adolescents (55). A previous meta-analysis on the effects of second-generation antidepressant therapy on attention and mental processing speed in patients with MDD suggested that SSRIs and dual inhibitors had the greatest positive effects on the processing speed of patients, and age, years of education, antidepressant treatment duration, and depressive status were major influencing factors (56). This is in line with the present study. A previous study in rats (57) showed that an injection of escitalopram (40–640 μg/kg i.v.) could directly increase DA release from the ventral tegmental area (VTA) and enhance DA transmission from the VTA to the frontal cortex, which directly improved executive function. A previous study also indicated that sigma-1 receptors were associated with various neurotransmitter systems and could affect their functions (58). Escitalopram was shown to enhance nerve growth factor-induced neurite growth in PC12 cells via sigma-1 receptor activation (59). In summary, improvements due to escitalopram in executive function in patients with MDD may be attributed to enhanced DA function through increases in DA release in the VTA and the activation of sigma-1 receptors. Therefore, the improvements in executive control networks assessed by the conflict RTs in the ANT may be related to escitalopram treatment. However, the mechanisms underlying the improvement in attention functions induced by escitalopram need further study, especially regarding long-term improvements in attention functions.

In the present study, patients who had poor sleep quality were administered a combined treatment of escitalopram and agomelatine. After 4 weeks of treatment, these patients showed better antidepressant and anxiolytic effects than those who took escitalopram alone. This difference, however, was no longer present in measurements after 8 and 12 weeks of treatment. Agomelatine, a melatonin receptor 1 and 2 agonist and 5-HT_2C_ antagonist, has unique antidepressant and anxiolytic mechanisms (60). Little is known about its effects on attention networks in patients with MDD. A previous study revealed that the mechanisms underlying the antidepressant effects of agomelatine were related to enhanced activity of the dopaminergic and adrenergic pathways projecting to the frontal cortex (60), and the mechanisms underlying the anxiolytic effects derived from the antagonism of 5-HT_2C_ receptors (61). Agomelatine heteromeric complexes of melatonin receptors MT1 and MT2 with 5-HT_2C_ receptors at the cellular level could translate into a synergistic action that can increase neuronal proliferation, maturation, and survival in the hippocampus through the modulation of multiple cellular pathways (i.e., increasing trophic factors, synaptic remodeling, and glutamate signaling) and key targets (i.e., immediate early genes and kinases) (60). The unique mechanisms of agomelatine may be related to the combination therapy being superior to escitalopram monotherapy after 4 weeks of treatment in the present study.

We found that escitalopram combined with agomelatine was superior to escitalopram monotherapy in improving the orienting RTs and not increasing the orienting ERs, with no correlation with changes in HAMD-17 and HAMA scores for the patients with MDD after 4 weeks of treatment. This suggests that cognitive dysfunction in patients with MDD may be independent of emotional symptoms (61). Agomelatine heteromeric complexes of MT1 and MT2 receptors with 5-HT_2C_ receptors at the cellular level could translate into synergistic actions that directly improve neuronal plasticity and resilience and increase neurogenesis in the hippocampus (62), which might contribute to improvements in the orienting network as measured by the ANT.

We also found that escitalopram alone and combined with agomelatine significantly improved the ANT conflict RTs after 4 weeks of treatment, and this improvement in the conflict RTs was not related to the improvement of emotional symptoms, which is in line with the previous study (63). A previous study reported that treatment with 10 and 20 mg/d of vortioxetine (64) could improve overall cognitive functions, including attention functions, compared with a placebo, independent of the alleviation of depressive symptoms (65), which is in line with the present findings. The present study found that the ANT miss rate was influenced by daytime functioning measured by the PSQI. No significant correlations were found between changes in the ANT RTs and changes in the total PSQI scores in the present study. This might be due to the use of subjective scales in evaluating sleep problems before and after treatment. Polysomnography should be used in future studies to objectively explore the association among sleep problems, attention networks, and specific symptoms in MDD.

### 4.1. Conclusions

Patients with moderate and severe MDD showed overall impairments of attention functions, including the three networks assessed by the ANT, namely the alerting, orienting, and executive control networks. Additionally, logical memory and subjective alertness were significantly impaired in patients with MDD. Treatment with both escitalopram alone and in combination with agomelatine could improve the RTs of the executive control network assessed by the ANT after 4 weeks of treatment, and this persisted until the end of the 12-week measurement period, but the levels did not recover to those of the HCs. Logical memory in patients with MDD was also improved after 4 weeks of treatment and recovered to the levels of the HCs after 8 weeks, lasting until the end of the 12-week treatment. Notably, combination with agomelatine was superior to escitalopram monotherapy regarding antidepressant and anxiolytic effects after 4 weeks. This was also true for the improvement of the orienting network RTs. The comorbidity of sleep problems in MDD might provide a useful scenario for exploring the relationship between specific depressive and anxious symptoms and attention impairments.

### 4.2. Limitations

There are some limitations to the present study. First, this was exploratory research using a single-blinded design, and there may be partial bias to some extent. Second, the sample size was relatively small and it was a pilot study. In the future, larger sample sizes should be used to carry out a randomized double-blind controlled study to further verify the present results.

## Data availability statement

The raw data supporting the conclusions of this article will be made available by the authors, without undue reservation.

## Ethics statement

The studies involving human participants were reviewed and approved by the Medical Ethics Committee of Peking University Sixth Hospital (Mental Health Institute) (Approval No 2016-12) and all subjects signed informed consent forms. The protocol was registered at clinicaltrials.gov with identification number NCT04978220. The patients/participants provided their written informed consent to participate in this study.

## Author contributions

X-QW, T-TW, and Y-FW designed the study. ZL, X-QW, T-TW, Y-TX, X-YZ, L-BG, and B-JH performed data acquisition and interpretation. Y-PB, X-QW, and S-XL performed the statistical analysis. ZL, X-QW, and S-XL wrote the manuscript. T-TW guided the ANT test application and data exploration. LL revised the manuscript for important intellectual content. All authors contributed to the study concept and reviewed and approved the final manuscript.

## References

[1] Fond G Micoulaud-Franchi JA Faugere M Boyer L Faget-Agius C Lançon Lançon C. Abnormal C-reactive protein blood levels as a specific biomarker of major depression and non-remission under antidepressants in schizophrenia. Prog Neuropsychopharmacol Biol Psychiatry. (2020) 97:109800. 10.1016/j.pnpbp.2019.109800 31676465

[2] Global Global burden of mental disorders the need for a comprehensive coordinated response from health social sectors at the country level: report by the Secretariat. World Health Organization. World Health Assembly. (2023) p. 65. Available online at: https://apps.who.int/iris/handle/10665/78898 (accessed May 27, 2023).

[3] Mendenhall E Kohrt BA Norris SA Ndetei D Prabhakaran D. Noncommunicable disease syndemics: poverty, depression, and diabetes among low-income populations. Lancet. (2017) 389:951–63. 10.1016/S0140-6736(17)30402-6 28271846 PMC5491333

[4] Nilsson J Thomas AJ Stevens LH McAllister-Williams RH Ferrier IN Gallagher P. The interrelationship between attentional and executive deficits in major depressive disorder. Acta Psychiatr Scand. (2016) 134:73–82. 10.1111/acps.12570 27037665

[5] Wang X Zhou H Zhu X. Attention deficits in adults with Major depressive disorder: a systematic review and meta-analysis. Asian J Psychiatr. (2020) 53:102359. 10.1016/j.ajp.2020.102359 32891927

[6] Keller AS Leikauf JE Holt-Gosselin B Staveland BR Williams LM. Paying attention to attention in depression. Transl Psychiatry. (2019) 9:279. 10.1038/s41398-019-0616-1 31699968 PMC6838308

[7] Sims A Oyebode F. SIMS' Symptoms in the Mind, 5th. Ed. Amsterdam: Elsevier Ltd. (2015).

[8] Zuckerman H Pan Z Park C Brietzke E Musial N Shariq AS. Recognition and treatment of cognitive dysfunction in major depressive disorder. Front Psychiatry. (2018) 9:655. 10.3389/fpsyt.2018.00655 30564155 PMC6288549

[9] Long Y Cao H Yan C Chen X Li L Castellanos FX. Altered resting-state dynamic functional brain networks in major depressive disorder: findings from the REST-meta-MDD consortium. NeuroImage Clinical. (2020) 26:102163. 10.1016/j.nicl.2020.102163 31953148 PMC7229351

[10] Posner M Fan J. Attention as an organ system. In:Pomerantz JR., editor. Topics in Integrative Neuroscience: From Cells to Cognition. Cambridge: Cambridge University Press (2008) p. 31–61. 10.1017/CBO9780511541681.005

[11] Petersen SE Posner MI. The attention system of the human brain: 20 years after. Annu Rev Neurosci. (2012) 35:73–89. 10.1146/annurev-neuro-062111-150525 22524787 PMC3413263

[12] Posner M Petersen SE. The attention system of the human brain. Annu Rev Neurosci. (1990) 13:25–42. 10.1146/annurev.ne.13.030190.000325 2183676

[13] Witte EA Marrocco RT. Alteration of brain noradrenergic activity in rhesus monkeys affects the alerting component of covert orienting. Psychopharmacology. (1997) 132:315–23. 10.1007/s002130050351 9298508

[14] Corbetta M Kincade JM Ollinger JM McAvoy MP Shulman GL. Voluntary orienting is dissociated from target detection in human posterior parietal cortex. Nature Neurosci. (2000) 3:292–97. 10.1038/73009 10700263

[15] Davidson MC Marrocco RT. Local infusion of scopolamine into intraparietal cortex slows covert orienting in rhesus monkeys. J Neurophysiol. (2000) 83:1536–49. 10.1152/jn.2000.83.3.1536 10712478

[16] Simon H Scatton B Moal ML. Dopaminergic A10 neurones are involved in cognitive functions. Nature. (1980) 286:150–51. 10.1038/286150a0 7402306

[17] Faraone SV Biederman J Spencer T Michelson D Adler L Reimherr F. Atomoxetine and stroop task performance in adult attention-deficit/hyperactivity disorder. J Child Adolesc Psychopharmacology. (2005) 15:664–70. 10.1089/cap.2005.15.664 16190797

[18] Mahableshwarkar AR Zajecka J Jacobson W Chen Y Keefe RS A. randomized, placebo-controlled, active-reference, double-blind, flexible-dose study of the efficacy of vortioxetine on cognitive function in major depressive disorder. Neuropsychopharmacology. (2015) 40:2025–37. 10.1038/npp.2015.52 25687662 PMC4839526

[19] Tian Y Du J Spagna A Mackie MA Gu X Dong Y. Venlafaxine treatment reduces the deficit of executive control of attention in patients with major depressive disorder. Sci Rep. (2016) 6:28028. 10.1038/srep28028 27306061 PMC4910055

[20] Shilyansky C Williams LM Gyurak A Harris A Usherwood T Etkin A. Effect of antidepressant treatment on cognitive impairments associated with depression: a randomized longitudinal study. The Lancet Psychiatry. (2016) 3:425–35. 10.1016/S2215-0366(16)00012-2 26995298 PMC4860142

[21] Luo LL Chen X Chai Y Li JH Zhang M Zhang JN. Distinct pattern of memory and attention deficiency in patients with depression. Chin Med J. (2013) 126:1144–49. 10.3760/cma.j.issn.0366-6999.20122636 23506595

[22] Cipriani A Furukawa TA Salanti G Chaimani A Atkinson LZ Ogawa Y. Comparative efficacy and acceptability of 21 antidepressant drugs for the acute treatment of adults with major depressive disorder: a systematic review and network meta-analysis. Focus. (2018) 16:420–29. 10.1176/appi.focus.16407 32021580 PMC6996085

[23] Herrera-Guzmán I Herrera-Abarca JE Gudayol-Ferré E Herrera-Guzmán D Gómez-Carbajal L Peña-Olvira M. Effects of selective serotonin reuptake and dual serotonergic-noradrenergic reuptake treatments on attention and executive functions in patients with major depressive disorder. Psychiatry Res. (2010) 177:323–29. 10.1016/j.psychres.2010.03.006 20385412

[24] Wroolie TE Williams KE Keller J Zappert LN Shelton SD Kenna HA. Mood and neuropsychological changes in women with midlife depression treated with escitalopram. J Clin Psychopharmacology. (2006) 26:361–66. 10.1097/01.jcp.0000227699.26375.f8 16855452

[25] Devroye C Cathala A Piazza PV Spampinato U. The central serotonin 2B receptor as a new pharmacological target for the treatment of dopamine-related neuropsychiatric disorders: rationale and current status of research. Pharmacol Ther. (2018) 181:143–55. 10.1016/j.pharmthera.2017.07.014 28757154

[26] Storbeck J Clore GL. How affective arousal influences judgments, learning, and memory. Soc Personal Psychol Compass. (2008) 2:1824–43. 10.1111/j.1751-9004.2008.00138.x 25067943 PMC4110743

[27] Pandi-Perumal SR Monti JM Burman D Karthikeyan R BaHammam AS Spence DW. Clarifying the role of sleep in depression: A narrative review. Psychiatry Res. (2020) 291:113239. 10.1016/j.psychres.2020.113239 32593854

[28] Cabanel N Schmidt AM Fockenberg S Brückmann KF Haag A Müller MJ. Evening preference and poor sleep independently affect attentional-executive functions in patients with depression. Psychiatry Res. (2019) 281:112533. 10.1016/j.psychres.2019.112533 31521842

[29] Shiga T Hoshino H Ochiai H Osakabe Y Kanno K Horikoshi S. Effects of benzodiazepine and orexin receptor antagonist on cognitive function revealed by auditory event-related potentials. J Psychopharmacol. (2021) 35:1488–95. 10.1177/02698811211035390 34330170

[30] Millan MJ. Multi-target strategies for the improved treatment of depressive states: conceptual foundations and neuronal substrates, drug discovery and therapeutic application. Pharmacol Ther. (2006) 110:135–70. 10.1016/j.pharmthera.2005.11.006 16522330

[31] Cardinali DP Srinivasan V Brzezinski A Brown GM. Melatonin and its analogs in insomnia and depression. J Pineal Res. (2012) 52:365–75. 10.1111/j.1600-079X.2011.00962.x 21951153

[32] American Psychiatric Association. Diagnostic and Statistical Manual of Mental Disorders, 5th ed. Washington, DC: American Psychiatric Association. (2013). 10.1176/appi.books.9780890425596

[33] Sheehan DV Lecrubier Y Sheehan KH Amorim P Janavs J Weiller E. et al. Mini International Neuropsychiatric Interview (MINI): the development and validation of a structured diagnostic psychiatric interview. J Clin Psychiatry. (1998) 59:22–33. 10.1037/t18597-000 9881538

[34] Reynolds KA Pankratz L Jain B Grocott B Bonin L King G. Moral injury among frontline long-term care staff and management during the COVID-19 pandemic. Front Health Serv. (2022) 2:841244. 10.3389/frhs.2022.841244 36925899 PMC10012813

[35] Fan J McCandliss BD Sommer T Raz A Posner MI. Testing the efficiency and independence of attentional networks. J Cogn Neurosci. (2002) 14:340–47. 10.1162/089892902317361886 11970796

[36] Rdsa A Afj A Rmk B. On the origins and evolution of the Attention Network Tests. Neurosci Biobehav Rev. (2021) 126:560–72. 10.1016/j.neubiorev.2021.02.028 33766674

[37] Psychology Software Tools. E-Prime Publications. (2023). Available online at: https://pstnet.com/e-prime-publications/

[38] Fan J Gu X Guise KG Liu X Fossella J Wang H. Testing the behavioral interaction and integration of attentional networks. Brain Cogn. (2009) 70:209–20. 10.1016/j.bandc.2009.02.002 19269079 PMC2674119

[39] Wechsler D. WMS-III: Wechsler Memory Scale Administration and Scoring Manual. San Antonio, TX: The Psychological Corporation. (1997).

[40] Shapiro CM Auch C Reimer M Kayumov L Heslegrave R Huterer N. A new approach to the construct of alertness. J Psychosom Res. (2006) 60:595–603. 10.1016/j.jpsychores.2006.04.012 16731234

[41] Lu L Wang XQ Tang XD. Sleep and Sleep Disorder Related Scale. Beijing: People's Medicine Publishing House. (2016). p. 469–471.

[42] Li S Fong DYT Wong JYH Wilkinson K Shapiro C Choi EPH. Psychometric evaluation of the Chinese version of the Toronto Hospital Alertness Test. J Patient Rep Outcomes. (2020) 4:32. 10.1186/s41687-020-00197-7 32372244 PMC7200959

[43] Fabre LF Smith LC. The effect of major depression on sexual function in women. J Sex Med. (2012) 9:231–9. 10.1111/j.1743-6109.2011.02445.x 21883948

[44] HAMILTON M A. rating scale for depression. J Neurol Neurosurg Psychiatry. (1960) 23:56–62. 10.1136/jnnp.23.1.56 14399272 PMC495331

[45] Maier W Buller R Philipp M Heuser I. The Hamilton Anxiety Scale: reliability, validity and sensitivity to change in anxiety and depressive disorders. J Affect Disord. (1988) 14:61–8. 10.1016/0165-0327(88)90072-9 2963053

[46] Buysse DJ Reynolds CF. 3rd, Monk TH, Berman SR, Kupfer DJ. The Pittsburgh Sleep Quality Index: a new instrument for psychiatric practice and research. Psychiatry Res. (1989) 28:193–213. 10.1016/0165-1781(89)90047-4 2748771

[47] Ringe WK Saine KC Lacritz LH Hynan LS Cullum CM. Dyadic short forms of the Wechsler Adult Intelligence Scale-III. Assessment. (2002) 9:254–60. 10.1177/1073191102009003004 12216782

[48] Spagna A He G Jin S Gao L Mackie MA Tian Y. Deficit of supramodal executive control of attention in schizophrenia. J Psychiatr Res. (2018) 97:22–9. 10.1016/j.jpsychires.2017.11.002 29172174

[49] Spagna A Mackie MA Fan J. Supramodal executive control of attention. Front Psychol. (2015) 6:65. 10.3389/fpsyg.2015.00065 25759674 PMC4338659

[50] Semkovska M Quinlivan L O'Grady T Johnson R Collins A O'Connor J. Cognitive function following a major depressive episode: a systematic review and meta-analysis. Lancet Psychiatry. (2019) 6:851–61. 10.1016/S2215-0366(19)30291-3 31422920

[51] Mendes T Cardoso S Guerreiro M Maroco J Silva D Alves L. Memory awareness in patients with Major Depressive Disorder. J Psychiatr Res. (2021) 137:411–18. 10.1016/j.jpsychires.2021.03.016 33774535

[52] Maeshima H Baba H Nakano Y Satomura E Namekawa Y Takebayashi N. Residual memory dysfunction in recurrent major depressive disorder–a longitudinal study from Juntendo University Mood Disorder Project. J Affect Disord. (2012) 143:84–8. 10.1016/j.jad.2012.05.033 22832170

[53] Escitalopram [Internet] National Library of Medicine; (2023). Available online at: https://www.ncbi.nlm.nih.gov/books/NBK557734/ (accessed May 27, 2023).

[54] Wang X Fan Y Li G Li H. The efficacy of escitalopram in major depressive disorder: a multicenter randomized, placebo-controlled double-blind study. Int Clin Psychopharmacol. (2021) 36:133–9. 10.1097/YIC.0000000000000350 33779577

[55] Choi CH Lee J Lee KH Hong SB Kim SH Han JY. Effects of antidepressant treatment on symptom measures of attention in adolescents with depression: a preliminary open-label study. J Child Adolesc Psychopharmacol. (2021) 31:288–93. 10.1089/cap.2020.0101 33417814

[56] Gudayol-Ferré E Duarte-Rosas P Peró-Cebollero M Guàrdia-Olmos J. The effect of second-generation antidepressant treatment on the attention and mental processing speed of patients with major depressive disorder: a meta-analysis study with structural equation models. Psychiatry Res. (2022) 314:114662. 10.1016/j.psychres.2022.114662 35689972

[57] Schilström B Konradsson-Geuken A Ivanov V Gertow J Feltmann K Marcus MM. Effects of S-citalopram, citalopram, and R-citalopram on the firing patterns of dopamine neurons in the ventral tegmental area, N-methyl-D-aspartate receptor-mediated transmission in the medial prefrontal cortex and cognitive function in the rat. Synapse. (2011) 65:357–67. 10.1002/syn.20853 20730799

[58] Bangaru ML Weihrauch D Tang QB Zoga V Hogan Q Wu HE. Sigma-1 receptor expression in sensory neurons and the effect of painful peripheral nerve injury. Mol Pain. (2013) 9:47. 10.1186/1744-8069-9-47 24015960 PMC3847629

[59] Albayrak Y Hashimoto K. Sigma-1 receptor agonists and their clinical implications in neuropsychiatric disorders. Adv Exp Med Biol. (2017) 964:153–61. 10.1007/978-3-319-50174-1_11 28315270

[60] Guardiola-Lemaitre B De Bodinat C Delagrange P Millan MJ Munoz C Mocaër E. Agomelatine: mechanism of action and pharmacological profile in relation to antidepressant properties. Br J Pharmacol. (2014) 171:3604–19. 10.1111/bph.12720 24724693 PMC4128060

[61] Pan Z Park C Brietzke E Zuckerman H Rong C Mansur RB. Cognitive impairment in major depressive disorder. CNS Spectr. (2019) 24:22–9. 10.1017/S1092852918001207 30468135

[62] Pompili M Serafini G Innamorati M Venturini P Fusar-Poli P Sher L. Agomelatine, a novel intriguing antidepressant option enhancing neuroplasticity: a critical review. World J Biol Psychiatry. (2013) 14:412–31. 10.3109/15622975.2013.765593 23530731

[63] Spagna A Wang J Rosario IE Zhang L Zu M Wang K. Cognitive considerations in major depression: evaluating the effects of pharmacotherapy and ECT on mood and executive control deficits. Brain Sci. (2022) 12:350. 10.3390/brainsci12030350 35326307 PMC8946784

[64] Gonda X Sharma SR Tarazi FI. Vortioxetine: a novel antidepressant for the treatment of major depressive disorder. Expert Opin Drug Discov. (2019) 14:81–9. 10.1080/17460441.2019.1546691 30457395

[65] McIntyre RS Lophaven S Olsen CK A. randomized, double-blind, placebo-controlled study of vortioxetine on cognitive function in depressed adults. Int J Neuropsychopharmcol. (2014) 17:1557–67. 10.1017/S1461145714000546 24787143 PMC4162519

