# Supplementary material for: A pilot study on improvements in attention function in major depressive disorder after 12 weeks of escitalopram monotherapy or combined treatment with agomelatine
Source: Front Psychiatry. 2023 Jun 22;14:1188175. doi: 10.3389/fpsyt.2023.1188175 (PMC10325661; doi:10.3389/fpsyt.2023.1188175)
Supplement: Supplementary file 1 [file Data_Sheet_1.docx]

# Supplementary Materials

Table S1. Demographic characteristics of MDD subgroups at baseline

| Variable | ESC (*n*=29) | | ESC+AGO (n=25) | | *t* **/ *χ²*** | *p* |
| --- | --- | --- | --- | --- | --- | --- |
|  | Mean | SD | Mean | SD |  |  |
| Age (years) | 31.19 | 9.84 | 31.72 | 9.19 | -0.20 | 0.844 |
| Gender |  |  |  |  |  |  |
| Male (n [%]) | 10 [38.46] | | 8 [32.00] | | 0.23 | 0.629*^b^* |
| Female (n [%]) | 16 [61.54] | | 17 [68.00] | |  |  |
| Education (years) | 14.23 | 3.43 | 15.68 | 3.41 | -1.51 | 0.137*^a^* |
| IQ | 118.11 | 14.38 | 123.16 | 15.18 | -1.22 | 0.229*^a^* |
| Recurrent MDE (n [%]) | 4 [15.38] | | 4 [16.00] | | 0.00 | 0.952*^b^* |
| Duration of the current MDE (months) | 5.52 | 4.90 | 6.52 | 4.69 | -0.76 | 0.448*^a^* |

# *^a^* Independent Samples t-test, *^b^* chi-square test. The values are expressed as numbers (%), mean ± standard deviation. MDD, major depressive disorder; ESC, escitalopram; AGO, agomelatine; SD, standard deviation; IQ, intelligence quotient; MDE, major depressive episode.

Table S2. Clinical assessments of MDD subgroups at baseline

| Variable | ESC (*n*=29) | | ESC+AGO (n=25) | | *t* | *p^a^* |
| --- | --- | --- | --- | --- | --- | --- |
|  | Mean | SD | Mean | SD |  |  |
| THAT | 14.34 | 7.75 | 11.60 | 5.65 | 1.44 | 0.156 |
| ZOGIM-A | 35.15 | 5.74 | 33.96 | 4.85 | 0.80 | 0.428 |
| DST | 13.73 | 3.53 | 14.88 | 3.07 | -1.23 | 0.222 |
| LMT | 3.92 | 2.01 | 6.80 | 4.96 | -2.69 | 0.011 |
| HAMD | 26.19 | 4.67 | 25.20 | 6.40 | 0.63 | 0.529 |
| HAMA | 22.53 | 5.47 | 22.40 | 6.94 | 0.07 | 0.937 |
| PSQI | 13.11 | 4.24 | 14.04 | 4.06 | -0.79 | 0.431 |

*^a^* Independent Samples t-test. The values are expressed as mean ± standard deviation. MDD, major depressive disorder; ESC, escitalopram; AGO, agomelatine; SD, standard deviation; THAT, Toronto Hospital Alertness Test; ZOGIM-A, Alertness Questionnaire; DST, digital span test; LMT, Logical Memory test; HAMD, Hamilton Depression Rating Scale; HAMA, Hamilton Anxiety Rating Scale; PSQI, Pittsburgh Sleep Quality Index.

Table S3. ANT parameters of MDD subgroups at baseline

| Variable | ESC (*n*=29) | | ESC+AGO (n=25) | | *Z* | *p^a^* |
| --- | --- | --- | --- | --- | --- | --- |
|  | Median | Quartile  (Q1, Q3) | Median | Quartile  (Q1, Q3) |  |  |
| RT (msec) |  |  |  |  |  |  |
| Alert | 43.77 | (32.70, 63.44) | 37.80 | (17.07, 61.11) | -0.91 | 0.361 |
| Orienting | 24.20 | (8.15, 56.73) | 18.54 | (-4.25, 42.91) | -1.26 | 0.207 |
| Conflict | 121.84 | (79.53, 147.01) | 112.19 | (79.40, 154.27) | -0.47 | 0.638 |
| Mean RT | 596.73 | (518.24, 755.51) | 624.93 | (553.41, 647.79) | -0.26 | 0.792 |
| ER (%) |  |  |  |  |  |  |
| Alert | 0.00 | (-4.17, 2.08) | 0.00 | (-2.08, 2.08) | -1.33 | 0.184 |
| Orienting | 0.87 | (-0.52, 2.60) | 0.00 | (0.00, 2.08) | -0.02 | 0.984 |
| Conflict | 2.78 | (1.04, 5.56) | 2.78 | (0.69, 4.86) | -0.66 | 0.511 |
| Mean RT | 1.74 | (0.69, 3.91) | 1.39 | (0.87, 3.30) | -0.38 | 0.705 |
| Miss rate | 0.00 | (0.00, 0.01) | 0.00 | (0.00, 0.01) | -0.29 | 0.772 |

^a^Mann-Whitney test. The values are expressed as medians, quartiles (Q1, Q3); MDD, major depressive disorder; ESC, escitalopram; AGO, agomelatine; ANT, Attention Network Test; RT, reaction time; ER, error rate.

Table S4. Clinical assessments of MDD subgroups after 4 weeks of treatment

| Variable | ESC (*n*=18) | | ESC+AGO (n=20) | | *t* | *p^a^* |
| --- | --- | --- | --- | --- | --- | --- |
|  | Mean | SD | Mean | SD |  |  |
| THAT | 20.33 | 7.15 | 19.05 | 8.39 | 0.51 | 0.617 |
| ZOGIM-A | 35.83 | 4.50 | 32.80 | 4.67 | 2.03 | 0.050 |
| DST | 15.44 | 2.55 | 15.20 | 3.35 | 0.25 | 0.803 |
| LMT | 2.39 | 2.33 | 3.85 | 3.20 | -1.62 | 0.114 |
| HAMD | 19.55 | 7.32 | 12.67 | 7.23 | 2.91 | 0.006 |
| HAMA | 18.40 | 7.21 | 13.06 | 6.51 | 2.40 | 0.022 |
| PSQI | 10.28 | 4.48 | 11.70 | 3.88 | -1.05 | 0.302 |

^a^Independent Samples t-test. The values are expressed as mean ± standard deviation. MDD, major depressive disorder; ESC, escitalopram; AGO, agomelatine; SD, standard deviation; THAT, Toronto Hospital Alertness Test; ZOGIM-A, Alertness Questionnaire; DST, digital span test; LMT, Logical Memory test; HAMD, Hamilton Depression Rating Scale; HAMA, Hamilton Anxiety Rating Scale; PSQI, Pittsburgh Sleep Quality Index.

Table S5. ANT parameters of MDD subgroups after 4 weeks of treatment

| Variable | ESC (*n*=18) | | ESC+AGO (n=20) | | *Z* | *p^a^* |
| --- | --- | --- | --- | --- | --- | --- |
|  | Median | Quartile  (Q1, Q3) | Median | Quartile  (Q1, Q3) |  |  |
| RT (msec) |  |  |  |  |  |  |
| Alert | 34.40 | (18.34, 43.95) | 50.82 | (24.02, 68.09) | -1.49 | 0.136 |
| Orienting | 27.97 | (13.02, 46.66) | 12.81 | (-8.93, 26.33) | -1.98 | 0.047 |
| Conflict | 87.52 | (68.61, 108.58) | 82.53 | (69.78, 106.83) | -0.23 | 0.815 |
| Mean RT | 507.84 | (491.26, 628.19) | 580.60 | (499.93, 617.73) | -0.93 | 0.349 |
| ER (%) |  |  |  |  |  |  |
| Alert | 0.00 | (-2.08, 2.08) | 0.00 | (-1.56, 2.08) | -0.45 | 0.649 |
| Orienting | 0.00 | (0.00, 2.08) | 0.00 | (-2.08, 2.08) | -0.23 | 0.818 |
| Conflict | 1.38 | (0.00, 2.77) | 2.08 | (0.34, 5.20) | -0.97 | 0.331 |
| Mean ER | 0.87 | (0.35, 1.30) | 1.39 | (0.69, 2.95) | -1.56 | 0.118 |
| Miss rate | 0.00 | (0.00, 0.00) | 0.00 | (0.00, 0.00) | -0.58 | 0.557 |

^a^Mann-Whitney test. The values are expressed as medians, quartiles (Q1, Q3); MDD, major depressive disorder; ESC, escitalopram; AGO, agomelatine; ANT, Attention Network Test; RT, reaction time; ER, error rate.
